# Psychometric properties of the Brisbane Burn Scar Impact Profile in adults with burn scars

**DOI:** 10.1371/journal.pone.0184452

**Published:** 2017-09-13

**Authors:** Zephanie Tyack, Roy Kimble, Steven McPhail, Anita Plaza, Megan Simons

**Affiliations:** 1 Centre for Children’s Burns and Trauma Research, Children’s Health Research Centre, The University of Queensland, South Brisbane, Queensland, Australia; 2 Institute of Health and Biomedical Innovation and School of Public Health and Social Work, Queensland University of Technology, Kelvin Grove, Queensland, Australia; 3 Centre for Functioning and Health Research, Metro South Health, Buranda, Australia; 4 Royal Brisbane and Women’s Hospital, Herston, Queensland, Australia; 5 Department of Occupational Therapy, Lady Cilento Children’s Hospital, South Brisbane, Queensland, Australia; BG Trauma Center Ludwigshafen, GERMANY

## Abstract

**Objective:**

The aim of the study was to determine the longitudinal validity, reproducibility, responsiveness and interpretability of the adult version of the Brisbane Burn Scar Impact Profile, a patient-report measure of health-related quality of life.

**Methods:**

A prospective longitudinal cohort study of patients with or at risk of burn scarring was conducted at three assessment points (at baseline around the time of wound healing, one to two weeks post-baseline and 1-month post-baseline). Participants attending a major metropolitan adult burn centre at baseline were recruited. Participants completed the Brisbane Burn Scar Impact Profile and the 36-item Short Form Health Survey and Patient Observer Scar Assessment Scale. Intraclass Correlation Coefficients (ICCs), smallest detectable change, percentage of those who improved, stayed the same or worsened and Area under the Receiver Operating Characteristic Curve (AUC) were used to test the aim.

**Results:**

Data were included for 118 participants at baseline, 68 participants at one to two weeks and 57 participants at 1-month post-baseline. All groups of items had acceptable reproducibility, except for the overall impact of burn scars (ICC = 0.69), the impact of sensations which was not expected to be stable (ICC = 0.63), mobility and daily activities (ICC = 0.63, 0.67 respectively). The responsiveness of six out of seven groups of items able to be tested against external criterion was supported (AUC = 0.72–0.75). Hypothesised correlations of changes in the Brisbane Burn Scar Impact Profile items with changes in criterion measures generally supported longitudinal validity (e.g., nine out of thirteen hypotheses using the SF-36 as an external criterion were supported). Internal consistency estimates, item-total and inter-item correlations indicated there was likely redundancy of some groups of items, particularly in the relationships and social interaction, appearance and emotional reactions items (Chronbach’s alpha range = 0.94–0.95).

**Conclusion:**

Support was found for the reproducibility, longitudinal validity, responsiveness and interpretability of most groups of Brisbane Burn Scar Impact Profile items and some individual items in the test population. Potential redundancy of items should be investigated further.

## Introduction

Burns can have devastating physical and psychological effects on individuals. In addition, treatments for burns and burn-related scarring use substantial amounts of healthcare resources [1] which, in the case of scar-related interventions, can continue long after the acute burn management has been completed. Scarring is a normal part of healing after tissue damage; however, in some patients an exaggerated response occurs involving complex molecular and cellular processes that are thought to be linked to immune [2] and sensorineural responses. The most common form of these exaggerated scar responses are hypertrophic scars [3] that are characterised by redness and thickness. It is estimated that 32 to 72 percent of patients with burns will develop hypertrophic scars [4]. The substantial burden of disease attributed to burn scars is demonstrated by findings that those with burn scarring have a significantly higher risk of re-admission and have treatment costs that are 5.6 times higher than those without scarring [5]. Studies of the effectiveness of scar interventions have been limited by the use of outcome measures with unknown psychometric properties among people with burn scarring and that have frequently not included the measurement of health-related quality of life.

The importance of measuring health-related quality of life in patients with burn scarring has been highlighted. Studies have found associations between burn scar severity, visibility and health-related quality of life [6], and limitations in three or more domains of health-related quality of life have been found in adults up to and including 18 months post-burn [7]. A new condition-specific patient-report outcome measure (PROM) of health-related quality of life was developed for people at risk of, or with burn scarring, as no such measure existed (termed the Brisbane Burn Scar Impact Profile—BBSIP, available at www.ccbtr.com.au [8]. This is the first known burn scar specific PROM measuring health-related quality of life. It is also the first multidimensional health-related PROM for people with burns to be developed based on interviews of patients themselves (N = 30 adults and children) [8], as existing burn-specific multidimensional health-related PROMs were developed using a literature review and expert opinion [9]. Content validity is also supported by the item development being based on a burn-scar specific conceptual framework of health-related quality of life [10]. Items were grouped into overall impact of scars; the intensity, frequency and impact of itch, pain and other sensations; work and daily activities (mobility and daily activities items); relationships and social interaction; and appearance, emotional reactions; and physical symptoms. The measure included items distinct from other burn-specific PROMs for burns including items measuring the intensity of tightness; sensitivity to cold, touch, or clothing; the impact of sensations when going downhill or downstairs; the impact of scar treatments and fatigue linked to burn scars; and the impact of scars on daily routines [8].

Although preliminary content validation has been conducted [8] work was required to test other psychometric properties of the Brisbane Burn Scar Impact Profile (BBSIP). The aim of this study was to test the longitudinal validity, reproducibility, and responsiveness of the BBSIP in the post-acute period in line with an evaluative purpose (tracking changes in scarring over this period).

## Materials and methods

### Design, setting, participants, and interventions

A prospective longitudinal cohort study of patients with three assessment points ((baseline around the time of wound healing, one to two weeks post-baseline (termed 1-week follow-up) and 1-month post-baseline (termed 1-month follow-up)) was conducted. The setting was an outpatient clinic that provided burn care to adults at a major metropolitan Australian hospital in Brisbane. Consecutive patients were sampled from 2013 to 2015 (17/04/2013–04/07/2013; 26/09/2013–10/10/2013; 16/01/2014–28/07/2014; 3/11/2014–24/11/2014) based on the availability of two assessors.

A sample size of 50 participants was sought for testing longitudinal validity, reproducibility, and responsiveness which is considered an appropriate sample size for testing those psychometric properties [11]. This sample size was sufficient to detect an ICC of 0.80 with 95% confidence intervals from 0.70 to 0.90 [12]. A sample size of 49 participants was also sufficient to detect a one sample correlation of 0.39 with 80 percent power and an alpha of 0.05 [13]. Eligibility criteria were: patients with scarring or with the potential to scar who attended the treating centre for their acute burn or follow-up management, aged over 18 years, having completed the BBSIP on at least one occasion, having burn wounds that were at least approximately 85% healed with the potential to scar at the baseline assessment. Patients with a range of burn scar severity (including those with scarring that restricted range of motion) were invited to participate. Patients with a cognitive, intellectual or physical impairment that impaired communication or memory were excluded, as were those with scars not caused by a burn injury and those who had eye or respiratory burns alone.

Participants received face-to-face usual care interventions during the study period which was tailored to their clinical presentation and the priorities identified by the treating team. Usual care in this setting included pressure garments, wound dressings, exercises, taping to prevent or manage contractures, oro-facial orthoses or splints to prevent or manage contractures, return-to-work programs, skin moisturizers, skin massage, medication for sleep, itch or psychopathology, psychological screening and non-pharmacological intervention such as counselling. The length and frequency of intervention received varied across participants. After skin healing outpatient appointments were typically scheduled weekly or fortnightly initially, if the patient required pressure garments that needed to be measured or fitted. Patients requiring ongoing scar management were typically reviewed monthly or second monthly if they lived locally or every three to six months if they lived a long distance from the treating centre (with regular follow-up continued locally). The majority of the participants received their acute burn care in the setting where the study was conducted.

### Questionnaires and other outcome measures

The adult version of the BBSIP, measuring health-related quality of life of adults with burns scars, was tested. The development of the measure, preliminary content validation and a conceptual framework linked to development of the measure have been previously described [8]. The measure consists of 66 items and 10 item groupings. Items of the BBSIP are rated using a range of response formats including dichotomous scales for items such as the presence or absence of open wounds; 11-point numeric rating scales for the intensity of sensory items (where 0 indicates the absence of the sensation and 10 indicates the sensation as bad as it can possibly be); 7-point likert scales for overall impact, daily living activities, emotional reactions, and social functioning; and 5-point likert scales for the frequency of sensory items and intensity of physical symptoms. The lower end of each scale indicates worse health-related quality of life.

Other measures included were the observer and patient scales of the Patient Observer Scar Assessment Scale (POSAS) and the 36-item Short Form Health Survey (SF-36, version 2.0). The POSAS has been deemed to have acceptable reliability but indeterminant content and construct validity and internal consistency in a 2012 systematic review of burn scar rating scales [14]. Since that review, further work has supported the internal consistency and predictive validity of the patient and observer scale [15]. Structural validity has generally been supported using RASCH analysis although inclusion of the surface area items when assessing burn scars was not supported [16] and thus was not included in this study. The SF-36 is a generic measure of health-related quality of life that consists of eight subscales that were used in this study (physical functioning, role-physical, bodily pain, general health, vitality, social functioning, role emotional and mental health). The eight SF-36 dimensions were scored on 0 to 100 percentage scales with lower values indicating worse health-related quality of life. The SF-36 has been validated in adult patients with burns with discriminant and temporal validity supported [17].

Demographic details and clinical characteristics were collected from patients or their medical records and included gender, highest level of education attained, scar location, restrictions in range of motion (or joint contractures or scars pulling on other body parts), skin grafting, age, Fitzpatrick skin type, percent total body surface area burned (TBSA), days post-burn, and days to wound healing.

### Procedures

The COnsensus based Standards for the selection of health Measurement INstruments (COSMIN) checklist [18] was used as a guide for reporting the psychometric properties. All outcome measures were completed in paper format at baseline, 1-week follow-up (to determine reproducibility) and 1-month follow-up (to determine longitudinal validity and responsiveness). The criteria of 85% skin healing for baseline measurements to be taken was judged visually by an assessor. Two assessors were involved in obtaining consent, providing instructions and the PROMs to participants. The order of the PROMs and instructions were standardised. A 1- to 2-week test retest interval was chosen as the period during which physical scar properties such as thickness and vascularity were expected to be relatively stable, based on other studies that have examined reproducibility of scar measures using this interval [19,20]. The POSAS and scar intensity components of the BBSIP were administered for the worst area of scarring, which patients were asked to identify prior to the measures being administered. The only exception to this was when the scar site was difficult to relocate. In this case another worst area of scarring was chosen by the patient. This worst area was identified using a 3 by 3cm area, marked on the skin using a soft tipped skin pencil. When patients were unable to return to the hospital to complete the measures, the 1-week follow-up and 1-month follow-up measures were posted with a self-addressed return envelope and instructions for completion at home. Approximate time to wound healing was obtained from the medical records or judged visually by the assessor when patients returned to the hospital.

### Statistical analysis

#### Validity (convergent and divergent)

Hypothesised correlations between scores on the BBSIP items and total score and respective items and subscale scores of the POSAS and SF-36 were specified a-priori (as highlighted in the tables) and were expected to be higher than 0.3 at baseline and higher than correlations with other items at 1-month follow-up. Correlations of changes in BBSIP scores with changes in respective SF-36 and POSAS items over the 1-month follow-up were also analysed to determine longitudinal validity. In brief, the pain item of the POSAS was expected to correlate more strongly with the pain item of the BBSIP as well as with the tightness item based on our previous work. The patient’s overall opinion of the scar on the POSAS was expected to correlate strongly with the appearance items of the BBSIP at baseline and for change from baseline to 1-month follow-up compared to respective correlations with other items. The sensory-related items of the BBSIP were expected to correlate more strongly with the itch and pain items of the POSAS than with other POSAS items, with correlations between respective items expected to be strongest (i.e. itch on the BBSIP with itch on the POSAS). Changes in BBSIP individual and group items related to mood and emotional responses were expected to correlate more strongly with the SF-36 mental health subscale than with other subscales. BBSIP individual and group items related to ADL, mobility, work and daily activities, and physical scar symptoms were expected to correlate more strongly with SF-36 physical functioning subscales than with other SF-36 subscales. BBSIP individual and group items related to social functioning were expected to correlate more strongly with the SF-36 social functioning subscale than with other SF-36 subscales. The BBSIP items of tight scars making you tired was expected to correlate more strongly with the vitality subscale of the SF-36 than with other SF-36 subscales.

#### Descriptive statistics, internal consistency and inter-item correlations

Descriptive statistics were used to describe the study sample (e.g. means and standard deviations, medians and interquartile ranges for continuous data, and frequencies and percentages for count data). Differences in demographic and clinical variables of participants between the time points were examined using related-sample data analyses (Wilcoxon Signed Rank Test for ordinal data and McNemar Test for nominal data). Missing data on the SF-36 data were imputed using the mean values of the remaining dimension items as recommended [21], when less than 50% of the items were missing. When 50% or greater of SF-36 subscale items were missing, data were treated as missing. Individual BBSIP items scored as not applicable were treated as missing and only complete data were used in individual item analyses. Total BBSIP subscale scores were calculated where the number of missing or not applicable items was less than 50% of the subscale items, by summing the available scores divided by the number of available items. Chronbach’s alpha and inter-item and item-total correlations using Spearman’s rho were used to indicate the internal consistency and inter-relatedness of items considered to group into sub-scales. Chronbach’s alpha of 0.7 to 0.9, item-total correlations of 0.3 to 0.7 [22] and inter-item correlations of approximately 0.20 to 0.40 [23] were considered ideal with higher values indicating potential redundancy of items.

#### Reproducibility and responsiveness

Reproducibility was tested by examining agreement and reliability. Agreement between baseline and 1-week follow-up was examined for all items using the number and percentage of items with exact agreement, agreement within 1-point and agreement within 2-points were also calculated for items with three or more response options. Agreement was also examined using standard errors of measurement (SEM), and smallest detectable change (SDC), where assumptions of normality were met. The SEM was calculated as √σ^2^ (where σ^2^ was the mean square error term from the ICC ANOVA) [11, 24] and the SDC was calculated as 1.96 x √2 x SEM. Reliability was examined using Cohen’s kappa for the dichotomous item and Intraclass Correlation Coefficients (ICC’s) for other items. ICC’s were calculated using a two-way, random effects model and associated confidence intervals and absolute agreement, with an ICC of greater than 0.7 considered acceptable [11].

Responsiveness was determined using Receiver Operating Characteristic Curves (ROC) to assess the area under the curve (AUC) corresponding to the correct identification of patients who changed using external criterion. The AUC can range from 0.5 (no discriminatory accuracy) to 1.0 (perfect accuracy) with an AUC of 0.7 indicating acceptable responsiveness of the item or domain [11]. Spearman’s rho was used to examine correlations between change scores and change in the external criterion with a correlation of 0.3 required for the external criterion to establish responsiveness [25]. Two comparisons were used where the POSAS patient scale items were an appropriate external criterion (worsening verses no worsening) and three comparisons were used where the SF-36 subscales were an appropriate external criterion (change versus no change), where change was defined as greater than the mean change per month on SF-36 subscales found in a previous study of an adult burn population [17] (e.g. greater than 5 points on the physical functioning scale versus less than or equal to 5 points).

#### Interpretability

It was assumed that evidence of some improvement would be detected in individual items and groups of items in the presence of tailored scar and psychosocial interventions that were typically provided after the baseline measurement, thus the percentage of those who stayed the same, improved and worsened was determined for the 1-month follow-up. At 1-month follow-up a greater number of participants were expected to have scar thickness and roughness stay the same or worsen than improve but for scar colour and sensory symptoms a larger number were expected to stay the same or improve compared to worsen, based on previous work on the trajectory of patient-reported scar severity in people receiving scar interventions similar to those provided to the study participants after burns [15]. Interpretability was also examined using the medians and interquartile ranges of subgroups expected to differ and percentage of missing items. Three subgroups of improved, stable and deteriorated were examined using SF-36 physical, social and mental health subscales and POSAS patient scale items of pain and overall opinion as criterion, with theory-based cutoffs used for the SF-36.

Both single items and groups of items of the BBSIP were tested where appropriate. Significance was reported using p-vales of less than 0.05. Statistical analyses were performed using IBM SPSS Statistics for Windows, Version 23.0. (Armonk, New York: IBM Corporation). Ethical approval was provided by Metro South Human Research Ethics Committee in 2011 (HREC12/QPAH/595) with written informed consent to participate obtained from all participants included in the study.

## Results

**Fig 1:**
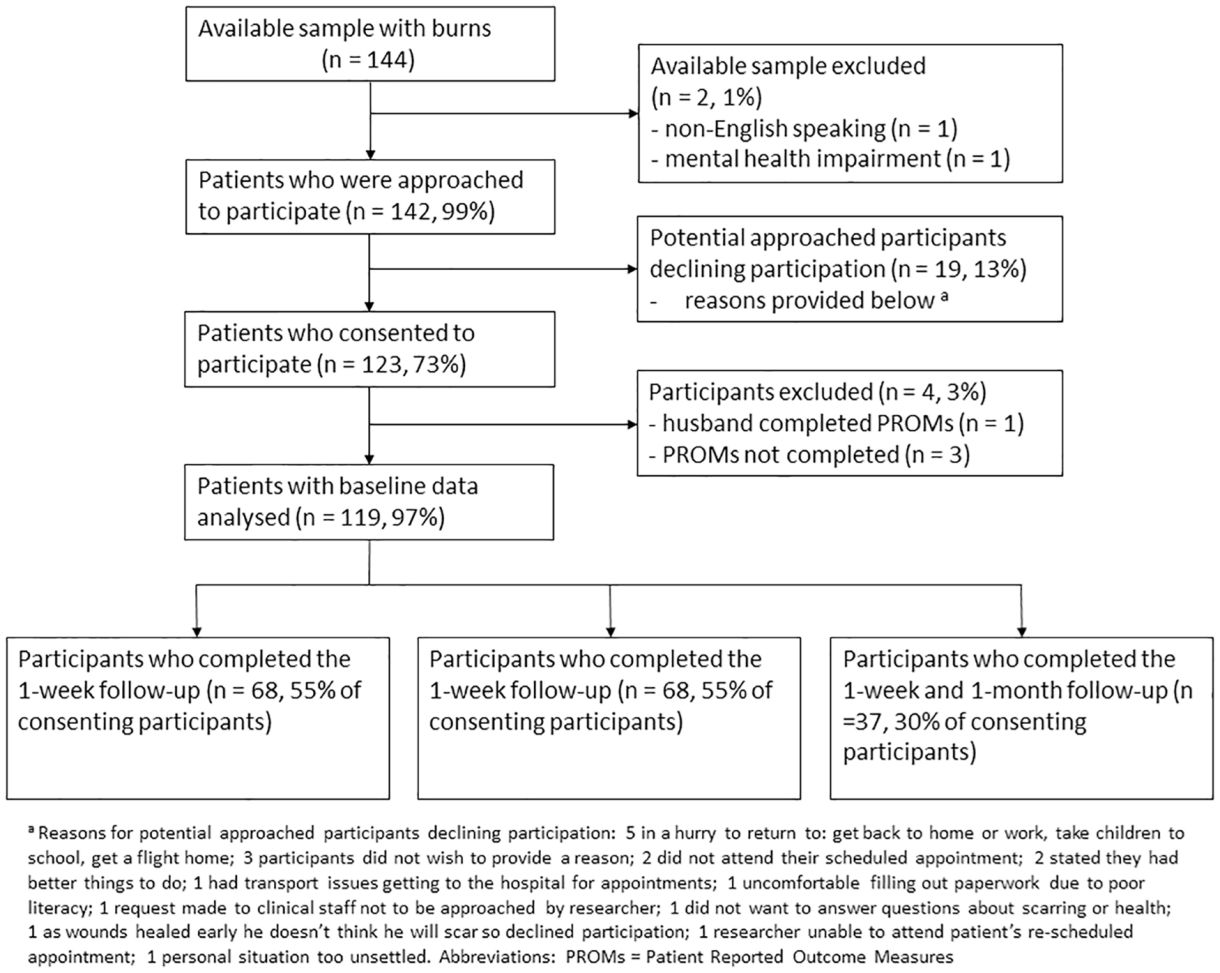
Flowchart of participants included and excluded.

The number of participants who were included in the study and completed testing at each timepoint is presented in Fig 1. Participant sociodemographic, injury, scar and skin type characteristics for the baseline, 1-week and 1-month follow-up samples are reported in Table 1. Participants were predominantly male, had a median age of 34 years, and the majority had a secondary education and received skin grafting. There were no significant differences in the characteristics of participants between baseline and 1-week follow-up and baseline and 1-month follow-up (Table 1).

**Table 1:** Participant sociodemographic, injury, scar and skin type characteristics at each testing timepoint.

Participant characteristics	Number of participants (% of sample)^a^		
	Baseline	1-week follow-up^b^	1-mth follow-up^b^
Number of participants (scars)	118 (118)	65 (65)	57 (57)
Male gender	87 (74%)	49 (75%)	42 (74%)
Education—highest level attained		(p = .32)^c^	(p = 1.00)^c^
Bachelor degree of higher (1)	15 (13%)	8 (12%)	4 (7%)
Diploma, Advanced diploma or post school certificate (2,3)	32 (27%)	20 (31%)	18 (32%)
Secondary education (4–7)	53 (45%)	29 (45%)	26 (46%)
Primary education or lower (8)	5 (4%)	3 (5%)	3 (5%)
Other (9,10)	2 (2%)	2 (3%)	2 (3%)
Missing	7 (6%)	2 (3%)	4 (7%)
Scar location			
Upper limbs	64 (54%)	35 (54%) (p = 1.00)	24 (42%) (p = 1.00)
Lower limbs	64 (54%)	23 (35%) (p = 1.00)	35 (61%) (p = 1.00)
Torso	30 (25%)	16 (25%) (p = 1.00)	18 (32%) (p = 1.00)
Head and neck	22 (19%)	13 (20%) (p = 1.00)	15 (26%) (p = 1.00)
Number of participants with restriction in range of motion or joint contractures or scars pulling on other body parts due to scarring	45 (38%)	24 (37%) (p = 1.00)	24 (42%) (p = 1.00)
Grafted	73 (62%)	45 (69%) (p = 1.00)	41 (72%) (p = 1.00)
Missing	3 (3%)	1 (2%)	0 (0%)
Median no. of skin grafts (interquartile range), range	1 (1), 0–16	1 (1), 0–16 (p = 1.00)	1 (2), 0–16 (p = 1.00)
Missing	3 (3%)	1 (2%)	0 (%)
Median age in years (interquartile range), range	34 (21), 18–85	34 (26), 18–80 (p = .32)	34 (22), 19–80 (p = 1.00)
Median Fitzpatrick skin type (interquartile range), range	3 (3–4), 1–5	3 (3–4), 1–5 (p = 0.32)	4 (3–4), 1–5 (p = 1.00)
Missing	1 (1%)	0 (0%)	0 (0%)
Median %TBSA burned (interquartile range), range	4 (1–10), <1–62	4 (1–15), <1–62 (p = .32)	7 (2–18), 1–62 (p = 1.00)
Missing	1 (1%)	1 (2%)	0 (0%)
Median days post-burn at baseline (interquartile range), range	27 (19–36), 7–150	26 (20–34), 9–150 (p = 1.00)	28 (22–41), 11–139 (p = 1.00)
Median days to wound healing (interquartile range), range	17 (9–27), 3–163	16 (7–30), 3–150 (p = .18)	22 (13–33), 3–163 (p = 1.00)
Missing	5 (4%)	2 (3%)	1 (2%)

^a^ % of sample in brackets except where indicated and no missing data except where indicated;

^b^ p values for participants at 1-week follow-up or 1-month follow-up compared to baseline;

^c^ scores 1–8 were included

Abbreviations: No. = number; %TBSA = percent Total Body Surface Area burned

At baseline the mean scar thickness score reported by an observer using the POSAS was 3.68 (SD = 2.00) which remained the same at 3.68 (SD = 2.03) at 1-month follow-up (scale range 1 to 10). At baseline the mean scar thickness reported by the patient using the POSAS was higher (mean = 5.82, SD = 2.77) and also remained relatively constant at 1-month follow-up (mean = 5.57, SD = 2.91). At baseline the mean overall opinion of the patient regarding their scar using the POSAS was 6.97 (SD = 2.33) which reduced slightly to 6.39 (SD = 2.43) at 1-month follow-up.

The percentage of missing scores for individual items varied from zero to three percent across the items at baseline and from zero to five percent across the items at 1-week follow-up (with the highest percent missing items on overall impact on life (5%) and driving a car or vehicle (3%)). At 1-month follow-up the percentage of missing items varied from zero to four percent across the items, with the highest percentage of missing items for appearance, emotional reactions and physical symptom items.

Cronbach’s alpha estimates as well as item-total and inter-item correlations (S1 Table) generally supported the internal consistency of subscales, but indicated there may be some redundancy of items within some item groupings, particularly for the relationships and social interaction items, appearance items and emotional reactions items.

Generally, hypothesised correlations of change in BBSIP items and groups of items with changes in criterion measures supported longitudinal validity (12 out of 19 hypotheses using the POSAS as the external criterion were supported and 9 out of 13 hypotheses using the SF-36 as an external criterion were supported). Correlations at baseline and between changes from baseline to 1-month follow-up on the BBSIP items and relevant POSAS patient scale items (Table 2) supported the validity and longitudinal validity of individual items of the overall impact of itch, pain and other sensations on your life, itch intensity, tightness intensity, sensitivity intensity, pain intensity, and tight and thick scar items; and the group items of the total sensory intensity score and physical symptoms total score. Correlations between changes from baseline to 1-month follow-up on the BBSIP items and relevant SF-36 dimensions (Table 3) supported the validity and longitudinal validity of the individual items of the overall impact on work and daily activities, overall impact on mood and emotional reactions, impact of sensations on physical scar symptoms and mood, and the physical scar symptom of ‘tight’. Validity and longitudinal validity was supported for the subscales of mobility, daily activities, social and relationships and emotional reactions.

**Table 2:** Correlations between BBSIP items and POSAS patient scale items at baseline and from baseline to 1-month follow-up corresponding to hypotheses.

BBSIP items (number of participants with data included in correlations)^a^	POSAS—patient scale items													
	Painful		Itching		Colour		Stiffness		Thickness		Irregular		Overall opinion compared to normal skin	
	T_0_	Δ	T_0_	Δ	T_0_	Δ	T_0_	Δ	T_0_	Δ	T_0_	Δ	T_0_	Δ
Item 2a Overall impact of itch, pain and other sensations on your life (n = 112–114; 49–52)	**0.48**	**0.38**	**0.59**	**0.35**	0.27	-0.26	0.28	0.07	0.31	0.02	0.31	-0.09	0.36	0.05
Item 8a Itch intensity (n = 112–114; 49–52)	0.29	0.19	**0.75**	**0.53**	0.23	0.08	0.14	0.01	0.24	0.10	0.25	0.01	0.29	0.11
Item 8b Tightness (n = 112–114; 49–52)	**0.41**	**0.26**	0.34	-0.13	0.26	-0.18	**0.49**	**0.15**	0.60	0.36	0.48	0.28	0.12	0.18
Item 8c Sensitivity intensity (n = 112–114; 49–52)	**0.48**	**0.14**	0.36	0.02	0.13	-0.08	0.16	0.01	0.30	0.14	0.25	0.01	0.12	-0.03
Item 8d Pain intensity (n = 112–114; 49–52)	**0.64**	**0.17**	0.25	-0.16	0.05	-0.17	0.33	0.04	0.26	-0.12	0.23	0.03	0.38	-0.14
Item 8e Discomfort intensity (n = 112–114; 49–52)	**0.62**	**0.22**	**0.32**	**0.06**	0.31	0.06	0.44	0.22	0.47	0.20	0.46	0.20	0.39	0.25
Total Sensory intensity score (n = 112–114; 49–52)	**0.58**	**0.24**	**0.49**	**0.07**	0.30	-0.06	0.41	0.11	0.52	0.16	0.46	0.16	0.27	0.09
Physical symptoms														
Item 17a Tight (n = 109–111; 47–50)	0.50	0.22	0.26	0.06	0.25	-0.06	**0.52**	**0.14**	0.47	0.08	0.38	0.15	0.36	0.13
Item 17b Thick (n = 109–111; 47–50)	0.36	0.22	0.32	0.07	0.21	-0.12	0.40	0.03	**0.57**	**0.29**	0.46	0.17	0.37	0.11
Item 17c Wrinkled (n = 109–111; 47–50)	0.31	0.09	0.11	-0.18	0.20	-0.16	0.32	0.05	0.39	0.21	**0.25**	**0.08**	0.22	0.18
Item 17e Hard (n = 109–111; 47–50)	0.32	0.22	0.11	-0.06	0.17	-0.30	0.41	0.07	**0.44**	**0.01**	0.30	-0.09	0.30	0.17
Item 17f Rough (n = 108–110; 47–50)	0.24	0.17	0.10	-0.15	0.19	-0.19	0.40	0.12	**0.54**	**0.20**	**0.41**	**0.05**	0.38	0.17
Item 17g Colour (n = 109–111; 47–50)	0.30	0.13	0.35	0.15	**0.54**	**0.01**	0.27	0.10	**0.31**	**0.03**	0.38	-0.06	0.47	0.13
Physical symptoms total score (n = 108–110; 47–50)	0.48	0.30	0.32	<0.01	**0.38**	**-0.24**	0.56	0.19	**0.62**	**0.18**	0.52	0.13	**0.49**	**0.30**
Item 14a The appearance of your scars (n = 111–113; 47–50)	0.36	0.13	0.16	0.25	0.30	0.11	0.31	-0.11	0.43	-0.08	0.45	-0.11	**0.42**	**0.16**
Item 14b Bothered by the look of the worst scar (n = 111–113; 47–50)	0.38	-0.06	0.20	0.20	0.28	0.02	0.31	-0.35	0.44	-0.26	0.48	-0.27	**0.45**	**-0.10**
Item 14c Bothered by looks you got from other people (n = 105–107; 45–47)	0.32	-0.07	0.17	0.14	0.21	-0.26	0.30	-0.12	0.42	-0.37	0.38	-0.26	**0.40**	**-0.02**
Item 14d Bothered by comments you got from other people (n = 105–107; 46–48)	0.22	-0.16	0.17	0.12	0.25	-0.07	0.23	-0.15	0.33	-0.27	0.32	-0.10	**0.37**	**-0.05**
Appearance total score (n = 104–106; 45–47)	0.34	-0.07	0.15	0.19	0.27	-0.05	0.31	-0.20	0.43	-0.28	0.42	-0.21	**0.44**	**0.04**

^a^ Spearman’s correlation coefficients;

T_0_ = baseline, Δ = change; correlations hypothesised to be strongest and positive are in bold

**Table 3:** Correlations between changes in BBSIP items and changes in SF-36 dimensions between baseline and 1-month follow-up^a^.

Changes in BBSIP items No. participants included in baseline correlations (No. in change correlations)	Baseline correlation (change correlation) in the SF-36 physical functioning dimensions				Baseline correlation (change correlation) in the SF-36 mental functioning dimensions			
	Physical functioning	Role physical	Bodily pain	General health	Vitality	Social functioning	Role emotional	Mental health
Item 3a Overall impact on work and daily activities n = 110–112 (n = 52–54)	**-0.35 (-0.41)**	**-0.44 (-0.39)**	-0.34 (-0.13)	-0.16 (0.14)	-0.30 (-0.24)	-0.34 (-0.23)	-0.27 (-0.13)	-0.25 (-0.21)
Item 3b Overall impact on social interactions or relationships n = 112–116 (n = 52–54)	-0.30 (-0.31)	-0.26 (-0.01)	-0.29 (0.12)	-0.10 (0.17)	-0.20 (0.23)	**-0.53 (-0.13)**	-0.26 (0.10)	-0.26 (0.16)
Item 3c Overall impact on mood or emotional reactions n = 112–119 (n = 52–54)	-0.35 (-0.38)	-0.28 (-0.16)	-0.38 (-0.05)	-0.26 (-0.04)	-0.41 (-0.06)	-0.55 (-0.19)	-0.41 (-0.34)	**-0.57 (-0.32)**
Impact of sensations on:								
Item 9c Physical scar symptoms n = 106–113 (n = 51–53)	**-0.26 (-0.50)**	-0.31 (-0.35)	-0.30 (-0.12)	-0.18 (-0.25)	-0.35 (-0.22)	-0.44 (-0.20)	-0.23 (0.05)	-0.30 (-0.32)
Item 9d Mood n = 112–119 (n = 52–54)	-0.32 (-0.32)	-0.26 (-0.22)	-0.35 (-0.08)	-0.31 (-0.23)	-0.47 (-0.09)	-0.48 (-0.24)	-0.43 (-0.23)	**-0.56 (-0.38)**
Mobility total score n = 111–115 (n = 51–54)	**-0.43 (-0.41)**	-0.30 (-0.07)	-0.48 (-0.16)	-0.33 (-0.11)	-0.50 (0.06)	0.48 (-0.05)	0.27 (0.23)	-0.34 (-0.10)
Daily activities total score n = 112-116(n = 51–54)	**-0.44 (-0.61)**	**-0.33 (-0.23)**	**-0.56 (-0.27)**	**-0.31 (-0.29)**	-0.46 (-0.26)	-0.48 (-0.35)	-0.31 (-0.05)	-0.40 (-0.24)
Appearance total score n = 111 = 115 (n = 47–49)	-0.25 (-0.14)	-0.20 (-0.06)	-0.44 (-0.09)	-0.22 (-0.21)	-0.38 (0.11)	**-0.49 (-0.08)**	-0.32 (-0.20)	-0.41 (0.12)
Social and relationships total score n = 112–116 (n = 50–52)	-0.46 (-0.47)	-0.37 (0.17)	-0.48 (-0.16)	-0.32 (-0.18)	-0.45 (0.07)	**-0.70 (-0.28)**	-0.40 (0.08)	-0.42 (-0.06)
Emotional reactions total score n = 111–115 (n = 48–51)	-0.27 (-0.30*)	-0.31 (-0.27)	-0.42 (-0.04)	-0.33 (-0.11)	-0.45 (0.04)	-0.58 (-0.23)	-0.48 (-0.14)	**-0.64 (-0.29)**
Physical scar symptoms								
Item 17a Tight n = 110–114 (n = 50–52)	**-0.39 (-0.28)**	-0.36 (-0.03)	-0.55 (-0.13)	-0.28 (-0.05)	-0.43 (0.03)	-0.38 (-0.10)	-0.21 (0.03)	-0.24 (-0.18)
Item 17b Thick n = 109–113 (n = 50–52)	**-0.19 (0.01)**	-0.25 (0.10)	-0.40 (0.10)	-0.26 (-0.04)	-0.38 (0.03)	-0.26 (-0.01)	-0.23 (-0.04)	-0.20 (<0.01)
Item 18 Tight scars making you tired n = 107–111 (n = 50–52)	-0.41 (-0.46)	-0.37 (-0.32)	-0.47 (-0.15)	-0.36 (-0.28)	**-0.54 (-0.26)**	-0.57 (-0.31)	-0.32 (-0.22)	-0.40 (-0.11)

^a^ Spearman’s rho correlations; correlations expected to be stronger than correlations with other SF-36 dimensions and negative are in bold

Abbreviations: no. = number

The longitudinal validity of individual items related to discomfort, colour, hardness and the appearance of scars was not supported using hypothesised correlations with changes in respective POSAS patient scale items, as correlations were stronger with changes in other POSAS items. However, the validity of the appearance items and appearance total score at baseline was supported. The correlations between changes in individual and group BBSIP items and changes in items of the SF-36 or POSAS were generally in the expected direction (negative correlations with the SF-36 = 16/17 correlations; positive correlations with the POSAS = 23/26 correlations).

### Reproducibility of the BBSIP, POSAS and SF-36

For the items expected to be stable, test-retest reliability coefficients were 0.6 or higher for the majority of items, and ranged from not acceptable (ICC = 0.26 for getting in and out of a chair or car) to acceptable (ICC = 0.80 for bothered by the appearance of scars) (Table 4). All of the individual appearance items had acceptable test-retest reliability based on the criterion of coefficients of 0.7 or higher. Most of the relationships and social interaction individual items had coefficients that were close to or higher than the criterion of 0.7 and thus mostly had acceptable test-retest reliability. Of the daily activities, mobility, or severity of physical symptom items only the eating and drinking item and the physical symptom total score had coefficients of 0.7 or higher. However the strength of ICC’s of the SF-36 physical functioning and role physical subscales (0.40 and 0.68) were similar to the daily activities and mobility item ICC’s (range 0.26 to 0.71). Further, the strength of physical symptom item ICC’s (0.39–0.77) had a similar range to ICC’s of corresponding POSAS-patient scale items (range 0.53 to 0.72) (S2 Table). Groups of items had higher test-retest reliability coefficients than individual items and were all greater than 0.7 except for the impact of sensations (which was not expected to be stable), overall impact of burn scars (which had an ICC that was close to acceptable), mobility and daily activities. Whilst reproducibility was not deemed a necessary property for items related to sensations and emotional responses by the investigators, the reliability of these items indicated they were almost as stable as other items with ICC values ranging from 0.47 (impact of sensations on physical activities) to 0.80 (embarrassed).

**Table 4:** Reproducibility of the BBSIP and POSAS individual items and BBSIP subscale scores using a one to two week retest period.

Items for the BBSIP ^a^	No of paired observations (no. of response scale points)	Intraclass correlation coefficient (95%CI)^b^	Residual	SEM	SDC ^c^	No. (%) of paired obs. with exact agreement	No. (%) of paired obs. ≥ 1 point difference	No. (%) of paired obs. ≥ 2 point difference
**Overall impact of burn scars**								
Item 1 Overall impact on life	61 (7)	0.54 (0.34–0.70)	1.29	1.14	3.16	19 (31%)	40 (65%)	54 (87%)
Item 2a Overall Impact of itch, pain and other sensations	65 (7)	0.52 (0.32–0.68)	1.26	1.12	3.10	15 (23%)	42 (65%)	60 (92%)
Item 2b Overall impact of physical scar symptoms	65 (7)	0.62 (0.44–0.75)	1.05	1.02	2.83	27 (42%)	47 (72%)	61 (94%)
Item 2c Impact of scar treatments	63 (7)	0.60 (0.42–0.74)	1.04	1.02	2.83	19 (30%)	45 (71%)	57 (90%)
Item 3a Impact on work and daily activities	65 (7)	0.39 (0.15–0.59)	1.88	1.37	3.80	15 (23%)	33 (51%)	50 (77%)
Item 3b Impact on social interaction or relationships	65 (7)	0.52 (0.33–0.68)	1.57	1.25	3.46	28 (43%)	49 (75%)	54 (83%)
Item 3c Impact on mood or emotional reactions	64 (7)	0.64 (0.46–0.76)	1.14	1.07	2.97	20 (31%)	42 (66%)	56 (88%)
Item 3d Impact on appearance	65 (7)	**0.71 (0.56–0.81)**	1.06	1.01	2.80	30 (46%)	53 (82%)	60 (92%)
Overall impact of burn scars total score	65 (7)	0.69 (0.50–0.81)	0.56	0.75	2.08	3 (5%)	47 (65%)	62 (95%)
**Frequency of sensory symptoms items**								
Item 5 Frequency of itch	64 (5)	0.58 (0.39–0.72)	0.69	0.83	2.30	22 (34%)	52 (81%)	61 (95%)
Item 6 Frequency of pain	64 (5)	0.76 (0.64–0.85)	0.45	0.67	1.86	30 (47%)	56 (88%)	64 (100%)
Item 7 Frequency of discomfort	64 (5)	0.66 (0.49–0.78)	0.69	0.83	2.29	30 (47%)	53 (83%)	61 (95%)
Frequency of sensory symptoms total score	64 (5)	**0.76 (0.63–0.85)**	0.31	0.56	1.55	13 (20%)	55 (80%)	64 (100%)
**Intensity of sensory symptoms items**								
Item 8a Itch intensity	65 (11)	0.69 (0.55–0.80)	2.55	1.60	4.43	15 (23%)	38 (58%)	51 (78%)
Item 8b Tightness intensity	65 (11)	**0.70 (0.55–0.80)**	2.58	1.61	4.46	16 (25%)	40 (62%)	54 (83%)
Item 8c Sensitivity intensity	65 (11)	0.60 (0.42–0.74)	2.95	1.72	4.76	20 (31%)	34 (52%)	49 (75%)
Item 8d Pain intensity	65 (11)	**0.70 (0.55–0.81)**	2.29	1.51	4.18	22 (34%)	45 (69%)	51 (78%)
Item 8e Discomfort intensity	65 (11)	0.67 (0.52–0.79)	2.62	1.57	4.35	18 (28%)	37 (57%)	51 (78%)
Item 20 Temperature sensitivity	65 (11)	0.56 (0.37–0.71)	4.07	2.02	5.60	14 (21%)	31 (48%)	44 (69%)
Intensity of sensory symptoms total score	65 (11)	**0.77 (0.66–0.86)**	1.16	1.08	2.99	6 (9%)	40 (62%)	54 (83%)
**Impact of sensations**								
Item 9a Impact of sensations on getting to sleep	65 (7)	0.66 (0.49–0.78)	1.02	1.00	2.77	25 (38%)	51 (78%)	55 (85%)
Item 9b Impact of sensations on staying asleep	65 (7)	0.53 (0.33–0.68)	1.41	1.19	3.29	27 (43%)	47 (72%)	53 (82%)
Item 9c Impact of sensations on physical activities	62 (7)	0.47 (0.27–0.64)	1.74	1.32	3.65	14 (23%)	42 (68%)	53 (85%)
Item 9d Impact of sensations on mood	65 (7)	0.48 (0.27–0.64)	1.65	1.28	3.55	20 (31%)	41 (63%)	53 (82%)
Item 9e Impact of sensations on walking downhill or downstairs	63 (7)	0.58 (0.39–0.72)	0.83	0.91	2.52	30 (48%)	51 (81%)	60 (95%)
Impact of sensations total score	65 (7)	0.63 (0.44–0.76)	0.63	0.79	2.19	10 (15%)	41 (63%)	59 (91%)
**Mobility and Daily Activities Items**								
Mobility items								
Item 10a Moving easily	64 (7)	0.68 (0.51–0.80)	0.86	0.93	2.57	28 (44%)	52 (81%)	58 (91%)
Item 10b Climbing up or down stairs	60 (7)	0.60 (0.41–0.74)	0.94	0.97	2.69	28 (47%)	48 (80%)	55 (92%)
Item 10c Getting in and out of a chair or car	60 (7)	0.26 (0.01–0.47)	1.00	1.00	2.77	31 (52%)	48 (80%)	52 (87%)
Item 10d getting in and out of a chair or car	62 (7)	0.59 (0.39–0.73)	0.86	0.93	2.58	28 (45%)	45 (73%)	56 (90%)
Mobility total score^c^	61 (7)	0.63 (0.45–0.76)	0.59	0.77	2.13	12 (20%)	48 (79%)	55 (90%)
**Daily activities items**								
Item 10e Driving a car or other vehicle								
Item 10f Physical activities	59 (7)	0.58 (0.38–0.73)	1.71	1.31	2.62	26 (44%)	41 (69%)	49 (83%)
Item 10g Work	47 (7)	0.57 (0.35–0.74)	2.08	1.44	3.99	17 (36%)	28 (60%)	36 (77%)
Item 10h Household activities	62 (7)	0.48 (0.24–0.66)	1.45	1.20	3.34	26 (42%)	43 (69%)	52 (84%)
Item 10i Dressing and undressing	63 (7)	0.63 (0.37–0.78)	0.93	0.96	2.66	23 (37%)	44 (70%)	56 (89%)
Item 10j Showering or bathing	58 (7)	0.50 (0.21–0.69)	1.32	1.15	3.18	23 (40%)	36 (62%)	45 (78%)
Item 10k Eating or drinking	63 (7)	**0.71 (0.57–0.82)**	0.60	0.77	2.15	36 (57%)	51 (81%)	60 (95%)
Item 10l Doing self-care activities	63 (7)	0.65 (0.47–0.48)	0.71	0.84	2.33	32 (51%)	53 (84%)	61 (97%)
Item 10m Activities that make you feel hot or sweaty	53 (7)	0.68 (0.50–0.80)	1.20	1.10	3.05	18 (33%)	35 (64%)	48 (87%)
Daily activities total score^c^	63 (7)	0.67 (0.42–0.81)	0.58	0.76	2.11	4 (6%)	38 (60%)	56 (89%)
Item 11a Daily routine	63 (7)	0.50 (0.13–0.72)	1.45	1.20	3.33	17 (27%)	39 (62%)	48 (76%)
Item 11b Family’s routine	58 (7)	0.61 (0.39–0.76)	1.65	1.28	3.56	16 (28%)	40 (69%)	48 (83%)
Item 12 Needing to change the way that you do work or daily activities	62 (7)	0.44 (0.15–0.64)	1.54	1.24	3.44	14 (23%)	37 (60%)	48 (77%)
**Relationships and social interaction Items**								
Item 13a Impact on doing things with friends	65 (7)	0.69 (0.50–0.81)	0.95	0.97	2.70	18 (28%)	49 (75%)	60 (92%)
Item 13b Impact on doing things with family	63 (7)	**0.72 (0.54–0.83)**	0.82	0.91	2.52	23 (37%)	49 (78%)	60 (95%)
Item 13c Impact on doing things with neighbours or relatives	60 (7)	0.69 (0.53–0.83)	0.93	0.96	2.66	27 (45%)	43 (72%)	57 (95%)
Item 13d Impact on interacting with the general public	63 (7)	**0.72 (0.56–0.82)**	0.82	0.91	2.52	23 (37%)	40 (63%)	55 (87%)
Item 13e Impact on close relationships	60 (7)	0.68 (0.50–0.79)	1.23	1.11	3.07	23 (38%)	42 (70%)	56 (93%)
Relationships and social interaction total score	60 (7)	**0.74 (0.56–0.85)**	0.63	0.79	2.19	11 (18%)	39 (65%)	57(95%)
**Appearance Items**								
Item 14a Bothered by the appearance of scars	64 (7)	**0.80 (0.69–0.87)**	0.79	0.89	2.47	27 (42%)	56 (86%)	61 (94%)
Item 14b Bothered by the look of the worst scar	65 (7)	**0.73 (0.60–0.83)**	1.13	1.06	2.95	26 (40%)	50 (77%)	58 (89%)
Item 14c Bothered by looks you got from other people	62 (7)	**0.75 (0.61–0.84)**	1.00	1.00	2.77	35 (56%)	54 (87%)	58 (94%)
Item 14d Bothered by comments you got from other people	62 (7)	**0.75 (0.61 0.84)**	0.92	0.96	2.66	31 (50%)	55 (89%)	60 (97%)
Appearance total score	63 (7)	**0.81 (0.71–0.88)**	0.65	0.81	2.25	16 (25%)	51 (81%)	60 (95%)
**Emotional Reactions Items**								
Item 15a Irritable or cranky	64 (7)	0.50 (0.29–0.67)	1.41	1.19	3.29	23 (36%)	45 (70%)	55 (86%)
Item 15b Anxious or nervous	64 (7)	0.63 (0.45–0.76)	1.00	1.00	2.77	31 (48%)	49 (77%)	57 (89%)
Item 15c Stressed	64 (7)	0.67 (0.51–0.79)	0.99	0.99	2.74	31 (48%)	49 (77%)	59 (92%)
Item 15d Depressed or sad	62 (7)	**0.79 (0.68–0.87)**	0.64	0.80	2.22	32 (52%)	53 (85%)	59 (95%)
Item 15e Angry	64 (7)	**0.70 (0.56–0.81)**	0.79	0.89	2.99	38 (59%)	55 (86%)	59 (92%)
Item 15f Low in self-confidence	62 (7)	0.66 (0.50–0.78)	1.05	1.02	2.84	35 (56%)	47 (75%)	59 (94%)
Item 15g Embarrassed	63 (7)	**0.80 (0.69–0.87)**	0.58	0.76	2.11	34 (54%)	56 (89%)	61 (97%)
Item 15h Worried	63 (7)	0.61 (0.43–0.75)	1.15	1.07	2.97	28 (44%)	45 (77%)	56 (89%)
Emotional Reactions total score	63 (7)	**0.77 (0.64–0.85)**	0.55	0.73	2.02	12 (19%)	47 (75%)	59 (94%)
**Severity of Physical Symptoms Items**								
Item 17a Tight scars	65 (5)	0.62 (0.44–0.75)	0.57	0.75	2.09	26 (40%)	55 (85%)	65 (100%)
Item 17b Thick scars	64 (5)	0.64 (0.46–0.76)	0.51	0.71	1.97	29 (45%)	56 (88%)	64 (100%)
Item 17c Wrinkled scars	64 (5)	0.62 (0.44–0.75)	0.44	0.66	1.83	34 (53%)	59 (92%)	63 (98%)
Item 17d Dry scars	63 (5)	0.39 (0.16–0.58)	0.82	0.94	2.61	24 (38%)	52 (81%)	60 (94%)
Item 17e Hard scars	63 (5)	0.51 (0.30–0.67)	0.61	0.78	2.16	31 (49%)	52 (83%)	61 (97%)
Item 17f Rough scars	63 (5)	0.56 (0.34–0.71)	0.52	0.72	2.00	38 (49%)	57 (92%)	59 (95%)
Item 17g Scars of a different colour than normal skin	64 (5)	0.57 (0.38–0.71)	0.57	0.75	2.08	26 (41%)	51 (80%)	64 (100%)
Physical symptoms total score	64 (5)	**0.77 (0.65–0.86)**	0.16	0.40	1.11	6 (9%)	60 (94%)	64 (100%)
Item 18 Tight scars resulting in tiredness	60 (7)	**0.74 (0.59–0.84)**	0.68	0.82	2.28	26 (43%)	46 (77%)	57 (95%)
Item 19^d^ Open wounds	64 (2)	0.34 (0.12)	N/A	N/A	N/A	N/A	N/A	N/A

^a^ Total scores were created using summed scores of individual items divided by the number of items in the scale. Where there were not applicable items the total score was obtained using an average of the remaining number of completed items.

^b^ Values of 0.7 or higher are in bold.

^c^ transformed using log 10 due to skewness

^d^ Cohen’s kappa with asymptotic standardised error has been reported for this dichotomous item

For the 7-point response scale items that were expected to be stable, agreement measured using the SDC ranged from 2.15 (eating or drinking) to 3.99 (work) and agreement within a 1-point difference ranged from 51 to 89 percent. For the 5-point response scale items that were expected to be stable (i.e. the severity of physical symptoms items), agreement measured using the SDC ranged from 1.83 to 2.61 and agreement within a 1-point difference ranged from 80 to 92 percent. The 11-point sensory intensity response scale items and the 7-point emotional response items were not expected to be stable thus reproducibility was not deemed a necessary property although values have been reported in Table 4.

### Responsiveness and longitudinal validity of the BBSIP

The number of participants included varied from 41 to 54 across ROC analyses, with greater than 50 participants included for the majority of analyses. The responsiveness of the individual item of overall impact on work and daily activities was supported using non-parametric estimations of the AUC (AUC>0.7). The responsiveness of seven out of eight subscales scores able to be tested against a criterion was supported including the frequency of sensory symptoms, impact of sensations, mobility, daily activities, relationships and social interaction, and physical symptoms (AUC> 0.7). The median BBSIP change scores of those who improved, stayed the same and deteriorated on the criterion were generally in the expected direction further supporting longitudinal validity (Table 5). The percentage of people who improved varied from 15 to 86 percent and who worsened varied from 2 to 38 percent across the BBSIP individual items and groups of items, from baseline to 1-month follow-up.

**Table 5:** Descriptive and AUC statistics for BBSIP items and subscale scores in people classified as stable, improved or worse on external criterion at 1-month follow-up.

Items for the BBSIP	Criterion	Improved^a^ Median (IQR) BBSIP change score n	Stayed the same^a^ Median (IQR) BBSIP change score n	Worsened^a^ Median (IQR) BBSIP change score n	AUC (95%CI)^b^
**Overall impact of burn scars**					
Item 2a Overall Impact of itch, pain and other sensations	POSAS-patient scale pain item (Spearman’s correlation coefficient between change scores = 0.38**)	1.00 (2.00) n = 31	1.00 (3.00) n = 7	0.50 (3.00) n = 14	0.68 (0.50–0.85) n = 52
Item 2b Overall impact of physical scar symptoms	SF-36 PF subscale (Spearman’s correlation coefficient between change scores = -0.17)	1.00 (1.00) n = 31	0.00 (2.50) n = 17	-1.00 (N/A) n = 3	N/A
Item 3a Impact on work and daily activities	SF-36 PF subscale (Spearman’s correlation coefficient between change scores = -0.41**)	2.00 (3.00) n = 33	1.00 (2.50) n = 17	0.00 (0.75) n = 4	**0.74 (0.60–0.88)** n = 54
Item 3b Impact on social interaction or relationships	SF-36 SF subscale (Spearman’s correlation coefficient between change scores = 0.22)	1.00 (2.00) n = 33	0.00 (0.75) n = 12	0.50 (2.50) n = 8	N/A
Item 3c Impact on mood or emotional reactions	SF-36 MH subscale (Spearman’s correlation coefficient between change scores = 0.47**)	1.00 (2.00) n = 30	1.00 (1.50) n = 9	0.00 (0.50) n = 13	0.41 (0.24–0.59) n = 52
Mobility total score	SF-36 PF subscale (Spearman’s correlation coefficient between with change scores of BBSIP = -0.38**)	0.75 (1.00) n = 33	0.00 (1.25) n = 17	0.13 (0.44) n = 4	**0.74 (0.59–0.89)** n = 54
ADL total score	SF-36 PF subscale (Spearman’s correlation coefficient between with change scores of BBSIP = -0.77**)	1.18 (1.45) n = 33	0.22 (1.12) n = 17	-0.06 (0.34) n = 4	**0.74 (0.61–0.88)** n = 54
Relationships and social interaction total score	SF-36 SF subscale (Spearman’s correlation coefficient with change score = -0.40**)	1.10 (2.16) n = 32	0.40 (0.47) n = 11	0.50 (2.40) n = 8	**0.73 (0.56–0.90)** n = 41
Appearance total score	SF-36 SF subscale (Spearman’s correlation coefficient between change scores = -0.08)	(5.25) n = 30	0.00 (2.00) n = 11	0.50 (4.50) n = 8	N/A
Emotional Reactions total score	SF-36 MH subscale (Spearman’s correlation coefficient between change scores = -0.32*)	0.25 (1.03) n = 30	0.25 (1.03) n = 8	0.00 (1.63) n = 13	0.40 (0.21–0.59) n = 50
Item 18 Tight scars resulting in tiredness	SF-36 Vitality subscale (Spearman’s correlation coefficient between change scores = -0.26)	0.00 (1.25) n = 30	0.5 (1.5) n = 10	0.00 (1.50) n = 12	N/A
Frequency of sensory symptoms total score	POSAS—patient scale pain item (0.66** Spearman’s correlation coefficient between change scores)	0.33 (1.00) n = 31	0.00 (0.67) n = 7	0.00 (1.33) n = 13	**0.72 (0.56–0.88)** n = 51
Intensity of sensory symptoms items total score	POSAS—patient scale pain item (Spearman’s correlation coefficient between change scores = 0.24)	0.60 (2.20) n = 31	0.60 (1.20) n = 7	0.00 (2.80) n = 14	N/A
Impact of sensations total score	SF-36 PF (Spearman’s correlation coefficient between change scores = 0.44**)	1.00 (0.80) n = 33	0.20 (1.20) n = 17	0.00 (2.55) n = 4	**0.72 (0.56–0.88)** n = 54
Physical symptoms total score	POSAS—patient scale overall opinion (Spearman’s correlation coefficient between change scores = 0.31*)	0.14 (0.57) n = 27	0.21 (1.25) n = 10	-0.29 (0.79) n = 10	**0.75 (0.59–0.75)** **n = 47**

Abbreviation: N/A = not applicable; where the correlation coefficient between change in the criterion and the BBSIP item or subscale was less than 0.3; AUC = Area under the Receiver Operating Characteristic Curve; POSAS = Patient Observer Scar Assessment Scale; PF = Physical Function; SF = Social Function; MH = Mental Health

^a^ For the SF-36 improved = improved functioning using the criterion cut-off (increase in subscale score), stayed the same = no change using the criterion cut-off, worsened = worse functioning using the criterion cut-off (decrease in subscale scores over time). Criterion cut-offs: SF-36 PF subscale (>5 points = improve/worsen, 5 to -5 points of change = stayed the same), SF-36 SF subscale (10 points or more = improve/worsen, <10 to -10 points = stayed the same), SF-36 MH subscale (5 points or more change = improve/worsen, <5 to -5 points = stayed the same), SF-36 V subscale (> 5 points = improve/worsen, 5 to -5 points = stayed the same). For the POSAS improved = any decrease in scores, stayed the same = no change in scores, worse = any increase in scores.

^b^ The dichotomous cut-offs for ROC curves were: SF-36 PF subscale = greater than 5 points indicating change versus 5 points or less of change indicating no change; SF-36 SF subscale = 10 points or more indicating change versus less than 10 points indicating no change; SF-36 Vitality subscale = greater than 5 points indicating change versus less than 5 points indicating no change; SF-36 MH subscale = 5 points or more indicating change versus less than 5 points indicating no change; POSAS patient scale items = no worsening versus worsening.

* = significance at the 0.05 level

** = significance at the 0.01 level

### Interpretability of the BBSIP

The percentage of individual items with the lowest score varied from 0 to 58 percent and for groups of items varied from 0 to 25 percent at baseline. The percentage of individual items with the highest score varied from 1 to 33 percent and for groups of items varied from 0 to 8 percent at baseline. High percentages of the lowest score were expected for many individual items as testing included people who were at risk of scarring who may have had few physical or sensory symptoms of scarring at baseline. As expected the majority of sensory items stayed the same or improved between baseline and 1-month follow-up. Changes in the physical symptom items were also as expected (e.g., scar thickness stayed the same or worsened for the majority of participants) over the same time period. The ability of the measure to detect change was demonstrated by improvement of up to 75 percent and by worsening of up to 36 percent on individual items scores between baseline and 1-month follow-up.

## Discussion

There was support for the longitudinal validity, reproducibility, responsiveness, and interpretability of most groups of items and some individual items of the BBSIP, thus the BBSIP appears suitable as an evaluative measure in people at risk of, or with burn scarring. However, not all results supported these properties, particularly the individual and group items related to mobility and daily activities, that were less reproducible than expected, and the appearance items for which baseline validity but not longitudinal validity was supported. In contrast, emotional and sensory symptoms items, that were expected to have reproducibility coefficients of lower than 0.7, performed better than expected. Support was also found for the validity of many items at baseline, although this was not the focus of the present study. It appears likely from the results that a shortened BBSIP may be appropriate for picking up change, based on the most responsive groups of items and likely redundancy of some items. However, further testing of the items at a longer time post-burn, and interpreting the results in the context of factor analysis is first recommended prior to deleting items or creating a shortened BBSIP that is suitable to pick up changes, which was beyond the scope of the present study but which is planned for a future study.

The BBSIP is the first known burn scar specific PROM measuring health-related quality of life, that was developed from interviews with patients themselves. As such it includes unique content in comparison to existing burn-specific multidimensional PROMs, by covering a broad range of sensory and emotional symptoms, fatigue and the impact of treatment linked to scarring. These existing PROMs include the Burn Specific Health Scale and shortened versions [26–28] and Young Adults Burns Outcome Questionnaire [29]. For example, the only sensory symptom captured by the brief and revised versions of the Burn-Specific Health Scale is heat sensitivity (not cold sensitivity, itch, pain, or sensitivity to light touch or clothing which are covered by the BBSIP). The only sensory symptoms captured by the Young Adults Burns Outcome Questionnaire are itch and pain in relation to the burned area (not in relation to the scar area) and the only emotional symptoms captured are being angry and sad (as opposed to the additional BBSIP symptoms of irritable or cranky, anxious or nervous, stressed, low in self-confidence, embarrassed and worried). Further, fatigue or vitality are not covered by the Burn-Specific Health Scale revised version or the Young Adults Burns Outcome Questionnaire. Although two items in the Abbreviated Burn Specific Health Scale cover this aspect these items are not specific to burn scars, thus differ to the BBSIP item of ‘tight scars resulting in tiredness’.

In comparison to existing multidimensional PROMs for patients with scars more broadly, the BBSIP also includes unique content. These existing PROMs include the Patient-Reported Impact of Scar Measure (PRISM) [19]; the Patient Scar Assessment Questionnaire (PSAQ) [30]; and Bock quality of life questionnaire for patients with keloid and hypertrophic scarring (Bock) [31]. For example, the PRISM does not include sensitivity to touch or clothing, sensitivity to hot or cold temperature, emotional symptoms of depression or anxiety, or scar tightness items which are represented in the BBSIP. Although the PSAQ and Bock each have an item representing pulling or stiffness [32], tightness is not specifically represented. In comparison, sensory or physical tightness is well represented in the BBSIP with 3-items, as it was highlighted as important based on our previous interviews with patients [10]. Interestingly, a similar sensory quality of ‘stiffness’ has been reported in other work involving patients with burn scars [33]. The importance of a ‘tightness’ quality is reflected by the likely action of myofibroblasts in creating contractile activity that results in tension in burn scars [34]. Scar contractures (which are present in up to 40 percent of people with burns) are the most severe form of this action [34].

Whilst the PRISM and the PSAQ had the advantage of being developed using interviews with patients [32], it is unclear whether the patients interviewed included any people with burn scars. No people with contracted scars were reported as being included in the development and validation samples of the PRISM [19]. This difference in populations may have contributed to the lack of items reflecting tightness in the PRISM. Further testing of the BBSIP and other scar-specific and burn-specific PROMs in future studies will be important to determine the relative importance of the unique content of the BBSIP in detecting patient-reported changes in burn scarring.

The test period, which commenced around the time of wound healing when exaggerated scar responses were emerging, may not have been the ideal period for reproducibility testing of mobility and daily activities items which were not stable. However, there is a need for a measure of health-related quality of life for people with burn scarring that can be used during periods when components of health-related quality of life are not stable. Therefore an alternative (more stable) point in the recovery (and/or less time between assessments) may be better for testing the reproducibility of these items. It is noteworthy that other PROMs that measured constructs similar to mobility and daily activity captured by the BBSIP (such as the SF-36 physical function and role physical subscales) had test-retest coefficients similar to the BBSIP in this study, supporting the likely instability of those components of health-related quality of life. Although Brown et al (2010) used a longer test-retest interval of at least 2-weeks for their quality of life measure for scars and reported reliability of 0.83 for the symptom scale and 0.89 for the quality of life scale, the lack of inclusion of people with contracted scars and testing that appeared to be conducted on scars with a mean duration of greater than 6-months likely contributed to differences in their findings [19].

The lower number of participants who completed follow-up testing in comparison to baseline was another important consideration when interpreting findings from the present study. It was plausible that there may have been variability in the characteristics of the samples between baseline and follow-up testing which may have impacted on the generalisability of the results. However, a comparison of the characteristics of participants between baseline and follow-up timepoints did not indicate that there were significant differences in the characteristics of the samples, which may indicate a lower likelihood of response bias influencing the results.

Future directions for testing the BBSIP include determining responsiveness using other external criterion such as the site of scarring which may have had a more consistent correlation of 0.3 or greater across BBSIP items. Interestingly the tightness and discomfort items correlated most strongly with the total score of the intensity of sensations subscale which may indicate further investigation of the method of obtaining a score for groups of items is warranted (i.e. a weighted score may be more appropriate than a summed score). However, Streiner and Norman (2015) have reported that in most cases a weighted score does not add substantially thus at this point using a simple summed score for groups of items would seem reasonable [22]. Testing using a larger sample size and over longer follow-up periods to confirm the validity of individual item and subscale change scores would be valuable as sample size and time between assessments may have impacted on the findings. Thus additional testing with larger-scale investigations, including the influence of subgroup differences such as those with skin contractures versus no skin contractures, are warranted in order to confirm or refute the favourable findings observed in the present study.

## Conclusions

Health-related quality of life should be an important focus of studies evaluating the effectiveness of scar interventions and of clinical rehabilitation of people at risk of or with burn scars. This study reports on the psychometric testing of the BBSIP with support for validity, longitudinal validity, reproducibility, responsiveness and interpretability of most groups of items and some individual items. As testing was conducted during the post-acute period (around the time of skin healing) when the greatest changes in health-related quality of life have been demonstrated, improved reproducibility and longitudinal validity estimates might reasonably be expected at a longer time post-burn.

## Supporting information

S1 Table Descriptive statistics and correlations of individual items of the BBSIP with subscale scores and respective SF-36 at baseline ^a^. (PDF)

S2 Table Reproducibility of the Patient Observer Scar Assessment Scale Items and SF-36 dimensions. (PDF)
