# Supplementary material for: Psychometric properties of the Brisbane Burn Scar Impact Profile in adults with burn scars
Source: PLoS One. 2017 Sep 13;12(9):e0184452. doi: 10.1371/journal.pone.0184452 (PMC5597202; doi:10.1371/journal.pone.0184452)
Supplement: S1 Table — (PDF) [file pone.0184452.s001.pdf]

S1 Table. Descriptive statistics and correlations of individual items of the BBSIP with subscale scores and respective SF-36 at baseline [a]

Items/ subscales of the BBSIP (n) [b]	Alpha	Item-total r_s	Potential range (actual score range where different) [c]	Median (IQR)	Range of inter-item correlations by subscale item [d]							
Overall disease burden (n = 119)	**0.87** (n=113)	n = 116-119	1-7 (1.4 – 6.9)	Mean (SD) = 3.6 (1.2)	1	2a	2b	2c	3a	3b	3c	3d
								(n = 114 – 119)				
Item 1 Life (n = 119)		0.81	1-7 (1-7)	4.0 (3.0)	-	0.45	0.55	**0.39**	0.44	0.63	0.61	0.57
Item 2a Itch, pain and other sensations (n = 119)		**0.65**	1-7 (2-6)	4 .0 (2.0)	0.45	-	0.56	0.47	**0.33**	**0.31**	**0.37**	**0.35**
Item 2b Physical scar symptoms (n = 119)		0.72	1-7 (1-5)	3.0 (2.5)	0.55	0.56	-	0.47	**0.34**	0.51	0.44	**0.39**
Item 2c Scar treatments (n = 119)		**0.64**	1-7 (2-6)	4.0 (2.0)	**0.39**	0.47	0.47	-	**0.28**	**0.38**	**0.36**	0.44
Item 3a Impact on work and daily activities (n = 119)		**0.63**	1-7 (2-7)	5.0 (3.0)	0.44	**0.33**	**0.34**	**0.28**	-	0.45	0.42	**0.33**
Item 3b Impact on social interaction or relationships (n = 119)		0.76	1-7 (1-5)	3.0 (3.0)	0.63	**0.31**	0.51	**0.36**	0.45	-	0.59	0.52

Item 3c Impact on mood or emotional reactions (n = 119)		0.79	1-7 (1-5)	3.0 (3.0)	0.61	**0.37**	0.44	0.44	0.42	0.59		0.69
Item 3d Impact on appearance (n = 119)		0.74	1-7	3 .0 (3.0)	0.57	0.35	0.39	0.42	**0.33**	0.52	0.69	-
**Frequency of sensory symptoms items (n = 118)**	**0.79** (n = 118)		1-5 (1-5)	Mean (SD) = 3.1 (1.1)	5a	6	7					
						(n = 118)						
Item 5 Frequency of itch (n = 118)		0.75	1-5 (1-5)	3.5 (2.0)	-	**0.37**	0.52					
Item 6 Frequency of pain (n = 118)		0.84	1-5 (1-5)	2.0 (2.0)	**0.37**	-	0.75					
Item 7 Frequency of discomfort (n = 118)		0.91	1-5 (1-5)	3.0 (2.0)	0.52	0.75	-					
**Intensity of sensory symptoms items (n = 119)**	**0.82 (n = 119)**		0 – 10 (0-8.2)	Mean (SD) = 3.8 (1.9)	8a	8b	8c	8d	8e	20		
							(n = 118-119)					
Item 8a Itch intensity (n = 119)		**0.63**	0-10 (0-10)	4.0 (4.0)	-	**0.37**	0.41	**0.33**	**0.34**	0.63		
Item 8b Tightness intensity (n = 119)		0.78	0-10 (0-10)	0.4 (0.5)	**0.37**	-	0.51	0.42	0.60	0.78		

Item 8c Sensitivity to touch/clothing intensity (n = 119)		0.78	0-10 (0-10)	3.0 (4.0)	0.41	0.51	-	0.56	0.60	0.78
Item 8d Pain intensity (n = 119)		**0.69**	0-10 (0-10)	2.0 (4.0)	**0.32**	0.42	0.56	-	0.69	0.69
Item 8e Discomfort intensity (n = 119)		0.81	0-10 (0-10)	3.0 (4.0)	**0.34**	0.60	0.60	0.69	-	0.81
Item 20 Temperature sensitivity (n = 119)		**0.66**	0-10	0.67	**0.33**	0.53	**0.34**	**0.22**	**0.38**	-
**Impact of sensations (n = 119)**	**0.79**<br>(n = 111)	n = 113-119	1 - 7 (1.0 – 6.8)	Mean (SD) = 3.2 (1.3)	9a	9b	9c	9d	9e	
							n = 113-119			
Item 9a Impact of sensations on getting to sleep (n = 119)		0.83	1-7, (1-7)	3.0 (2.0)	-	0.74	**0.46**	**0.46**	**0.40**	
Item 9b Impact of sensations on staying asleep (n = 119)		0.73	1-7 (1-7)	2.0 (3.0)	0.74	-	**0.32**	**0.36**	**0.38**	
Item 9c Impact of sensations on physical activities (n = 113)		0.75	1-7 (1-7)	4.0 (3.0)	0.46	**0.32**	-	0.45	**0.35**	

Item 9d Impact of activities on mood (n = 119)		0.71	1-7 (1-7)	3.0 (2.0)	0.46	**0.36**	0.45	-	**0.30**
Item 9e Impact of sensations on walking downhill or downstairs (n = 117)		**0.61**	1-7 (1-7)	2.0 (2.0)	**0.40**	**0.38**	**0.35**	**0.30**	-
**Mobility and Daily Activities Items (n = 111)**									
Mobility items (n = 119)	**0.88**<br>(n = 111)		1-7 (1-6.5)	Median = 2.3 (IQR = 2.0)	10a<br>(n = 113 – 118)	10b	10c	10d	
Item 10a moving easily (n = 118)		0.88	1-7 (1-7)	3.0 (2.0)	-	0.63	0.61	0.68	
Item 10b Climbing up or down stairs (n = 113)		0.85	1-7 (1-7)	2.0 (2.0)	0.63	-	0.78	0.62	
Item 10c Walking short distances (n = 114)		0.86	1-7 (1-5)	1.0 (2.0)	0.61	0.78	-	0.78	
Item 10d getting in and out of a chair or car (n = 117)		0.87	1-7 (1-7)	2.0 (2.0)	0.68	0.62	0.78	-	

Daily activities items (n = 119)	**0.89 (**n = 78)		1-7 (1-7)	Mean (SD) = 3.3 (1.5)	10e	10f	10g	10h	10i	10j	10k	10l	10m
									(n = 82 – 118)				
Item 10e (n = 103)		0.83	1-7 (1-7)	2.0 (3.0)	-	0.70	0.75	0.63	0.46	0.47	0.43	0.49	0.59
Item 10f Physical activities (n = 109)		0.73	1-7 (1-7)	4.0 (2.1)	0.70	-	0.51	0.49	0.43	0.36	**0.28**	**0.33**	0.61
Item 10g Work (n = 89)		0.80	1-7 (1-7)	5.0 (4.5)	0.75	0.51	-	0.71	0.40	0.53	0.42	0.45	0.43
Item 10h Household activities (n = 117)		0.81	1-7 (1-7)	4.0 (3.0)	0.63	0.49	0.71	-	0.58	0.64	0.56	0.62	0.51
Item 10i Dressing and undressing (n = 118)		0.76	1-7 (1-7)	3.0 (2.0)	0.46	0.43	**0.40**	0.58	-	0.69	0.71	0.60	0.62
Item 10j Showering or bathing (n = 118)		0.74	1-7 (1-7)	3.0 (3.0)	0.47	**0.36**	0.53	0.64	0.71	-	0.57	0.61	0.39
Item 10k Eating or drinking (n = 118)		**0.69**	1-7 (1-7)	1.0 (2.0)	0.43	**0.28**	0.42	0.56	0.60	0.57	-	0.71	0.44
Item 10l Doing self-care activities (n = 117)		0.73	1-7 (1-7)	1.0 (2.0)	0.49	**0.33**	0.45	0.62	0.62	0.61	0.71	-	0.56
Item 10m Activities that make you feel hot or sweaty (n = 103)		0.71	1-7 (1-7)	3.0 (4.0)	0.59	0.61	0.43	0.45	0.51	**0.39**	0.44	0.56	-

Impact on routine (n = 119)	**0.78** (n = 103)		1-7 (1-7)	Mean (SD) = 4.1 (1.6)	11a	11b	12		
					n = 103 - 112				
Item 11a Daily routine (n = 114)		0.87	1-7 (1-7)	5.0 (3.0)	-	0.54	0.66		
Item 11b Family's routine (n = 106)		0.81	1-7 (1-7)	3.0 (4.0)	0.51	-	0.44		
Item 12 Needing to change the way that you do work or daily activities (n = 117)		0.81	1-7 (1-7)	5.0 (3.0)	0.66	0.44	-		
**Relationships and social interaction Items (n = 119)**	0.95 (n = 103)		1-7 (1-6.8)	Median (IQR) = 2.8 (2.2)	13a	13b	13c	13d	13e
							n = 104 - 109		
Item 13a Impact on doing things with friends (n = 119)		0.88	1-7 (1-7)	3.0 (2.0)	-	0.84	0.77	0.68	0.75
Item 13b Impact on doing things with family (n = 115)		0.94	1-7 (1-7)	3.0 (3.0)	0.84	-	0.85	0.82	0.75
Item 13c Impact on doing things with		0.91	1-7 (1-7)	2.0 (3.0)	0.77	0.85	-	0.82	0.70

neighbours or relatives (n = 112)									
Item 13d Impact on interacting with the general public (n = 117)		0.88	1-7 (1-7)	3.0 (3.0)	0.69	0.82	0.82	-	0.67
Item 13e Impact on close relationships (n = 108)		0.87	1-7 (1-7)	3.0 (3.8)	0.72	0.76	0.71	0.70	-
**Appearance Items (n = 111)**	0.95 (n = 111)	n = 112 - 118	1-7 (1-7)	Median (IQR) = 2.5 (3.0)	14a	14b	14c	14d	
						n = 111 - 118			
Item 14a Bothered by the appearance of scars (n = 118)		0.95	1-7 (1-7)	3.0 (3.0)	-	0.96	0.85	0.75	
Item 14b Bothered by the look of the worst scar (n = 118)		0.95	1-7 (1-7)	3.0 (3.3)	0.96	-	0.84	0.75	
Item 14c Bothered by looks you got from other people (n = 118)		0.94	1-7 (1-7)	2.0 (3.0)	0.85	0.84	-	0.88	
Item 14d Bothered by comments you got from other people (n = 118)		0.88	1-7 (1-7)	2.0 (2.8)	0.75	0.75	0.88	-	

**Emotional reactions items (n = 118)**	0.94<br>(n = 113)	n = 117-118	1-7 (1-6.1)	Median (IQR) = 2.0 (1.9)	15a	15b	15c	15d	15e	15f	15g	15h
									n = 116 - 118			
15Item 15a Irritable or cranky (n = 118)		0.82	1-7 (1-7)	2.0 (2.0)	-	0.70	0.73	0.70	0.70	0.65	0.51	0.60
Item 15b Anxious or nervous (n = 117)		0.80	1-7 (1-7)	2.0 (2.0)	0.70	-	0.75	0.67	0.60	0.71	0.55	0.65
Item 15c Stressed (n = 117)		0.89	1-7 (1-7)	2.0 (3.0)	0.73	0.75	-	0.81	0.71	0.80	0.63	0.75
Item 15d Depressed or sad (n = 117)		0.89	1-7 (1-7)	2.0 (2.0)	0.70	0.67	0.81	-	0.74	0.85	0.69	0.70
Item 15e Angry (n = 117)		0.81	1-7 (1-7)	1.0 (2.0)	0.70	0.60	0.71	0.74	-	0.72	0.60	0.65
Item 15f Low in self-confidence (n = 117)		0.89	1-7 (1-7)	2.0 (2.0)	0.65	0.71	0.80	0.85	0.72	-	0.73	0.77
Item 15g Embarrassed (n = 118)		0.77	1-7 (1-7)	2.0 (2.0)	0.51	0.55	0.63	0.69	0.60	0.73	-	0.67
Item 15h Worried (n = 118)		0.86	1-7	2.0 (2.0)	0.60	0.65	0.75	0.70	0.65	0.77	0.67	-
**Severity of Physical Symptoms Items**<br>**(n = 116)**	**0.83**<br>(n = 115)	n = 115-116	1-5<br>(1.1 – 4.7)	Median (IQR) = 2.2 (1.0)	17a	17b	17c	17d	17e	17f	17g	
								n = 115-117				

Item 17a Tight scars (n = 116)		0.77	1-5 (1-5)	2.0 (1.0)	-	0.62	0.48	**0.38**	0.41	**0.33**	**0.33**
Item 17b Thick scars (n = 115)		0.78	1-5 (1-5)	2.0 (2.0)	0.62	-	0.51	**0.29**	0.53	0.52	**0.25**
Item 17c Wrinkled scars (n = 115)		**0.67**	1-5 (1-5)	2.0 (1.0)	0.48	0.51	-	**0.24**	**0.40**	0.58	0.16
Item 17d Dry scars (n = 115)		**0.56**	1-5 (1-5)	2.0 (1.0)	**0.38**	**0.29**	**0.24**	-	0.42	**0.29**	0.14
Item 17e Hard scars (n = 115)		**0.65**	1-5 (1-5)	2.0 (1.0)	0.41	0.53	**0.40**	0.42	-	0.52	0.11
Item 17f Rough scars (n = 114)		**0.68**	1-5 (1-5)	2.0 (2.0)	**0.33**	0.52	0.58	**0.29**	0.52	-	0.16
Item 17g Scars of a different colour than normal skin (n = 115)		**0.50**	1-5 (1-5)	4.0 (2.0)	**0.33**	**0.25**	0.16	0.14	0.11	0.16	-
Item 18 Tight scars resulting in tiredness (n = 114)	n/a	n/a	1-7 (1-7)	2 (2)	n/a	n/a	n/a	n/a	n/a	n/a	
Item 19 Presence of open wounds (n = 118)	n/a	n/a	0-1 (0-1)	Yes = 71 (60%)	n/a	n/a	n/a	n/a	n/a	n/a	

[a] Values in the ideal range are in bold

[b] Number of participants for the item-total correlations, potential score range and median (IQR) except where indicated

[c] Total scores were summed scores of individual items divided by the number of applicable items as some items could be scored not applicable

[d] Spearman's rho

Abbreviations: n/a = not applicable
