# Supplementary material for: Psychometric properties of the Brisbane Burn Scar Impact Profile in adults with burn scars
Source: PLoS One. 2017 Sep 13;12(9):e0184452. doi: 10.1371/journal.pone.0184452 (PMC5597202; doi:10.1371/journal.pone.0184452)
Supplement: S2 Table — (PDF) [file pone.0184452.s002.pdf]

S2 File. Reproducibility of the Patient Observer Scar Assessment Scale Items and SF-36 dimensions

Scale	No of paired obs. (no. of response option points)	Intraclass correlation coefficient (95%CI)	Residual	SEM	SDC	No. (%) of paired observations with exact agreement	No. (%) of paired observations with exact agreement and 1 point difference	No. (%) of paired observations with exact agreement, 1 or 2 point difference
POSAS – patient scale								
Item 1 Painful	59 (10)	0.72 (0.51 -0.84)	1.66	1.29	3.58	18 (31%)	39 (66%)	49 (83%)
Item 2 Itching	59 (10)	0.53 (0.29 – 0.70)	3.03	1.74	4.82	9 (15%)	27 (46%)	43 (73%)
Item 3 Colour	59 (10)	0.67 (0.47 – 0.80	1.62	1.27	3.53	19 (32%)	35 (59%)	49 (83%)
Item 4 Stiffness	59 (10)	0.65 (0.48 – 0.78)	2.72	1.65	4.57	16 (2%)	33 (43%)	45 (76%)
Item 5 Thickness	59 (10)	0.57 (0.36 – 0.72)	3.34	1.83	5.07	13 (22%)	36 (61%)	53 (90%)
Item 6 Irregular	58 (10)	0.72 (0.57 – 0.83	2.10	1.45	4.02	17 (29%)	33 (57%)	48 (83%)
Item 7 overall appearance	60 (10)	0.65 (0.48 – 0.78)	2.26	1.50	4.16	19 (32%)	32 (53%)	49 (82%)
POSAS – observer scale								
Item 1 Vascularity	49 (10)	0.63 (0.42 – 0.77)	1.59	1.26	4.41	15 (31%)	31 (63%)	40 (82%)
Item 2 Pigmentation	49 (10)	0.85 (0.75 – 0.92)	0.99	0.99	2.74	22 (45%))	39 (80%)	45 (92%)

Item 3 Thickness [a]	48 (10)	0.78 (0.64 – 0.87)	0.93	0.96	2.67	17 (35%)	39 81%	45 (94%)
Item 4 Relief	49 (10)	0.60 (0.39 – 0.76)	2.13	1.46	5.90	11 (22%)	35 (71%)	40(82%)
Item 5 Pliability	48 (10)	0.85 (0.74 – 0.92)	1.00	1.0	2.77	17(35%)	37(77%)	44 (92%)
Item 7 Overall opinion [a]	48 (10)	0.78 (0.63 – 0.87)	0.87	0.93	2.58	13 (27%)	35(73%)	45 (94%)
SF-36[b]								
Physical Function	61 (3)	0.40 (0.16 – 0.59)	280.21	16.74	46.40	11 (18%)	21 (34%)	28 (46%)
Role Physical [c]	58 (5)	0.68 (0.51 – 0.80)	292.12	17.09	47.37	8 (14%)	19 (33%)	19 (33%)
Bodily Pain	59 (5,6)	0.53 (0.29 – 0.70)	324.60	18.02	49.95	13 (22%)	13 (22%)	24 (41%)
General Health	59 (5)	0.78 (0.65 – 0.86)	74.06	8.61	23.87	16 (27%)	30(51%)	42 (71%)
Vitality	60 (5)	0.59 (0.40 – 0.73)	192.91	13.89	38.50	12 (20%)	26 (43%)	26 (43%)
Social Function	60 (5)	0.71 (0.55 -0.82)	210.54	14.51	40.22	18 (30%)	18 (30%)	18 (30%)
Role Emotional	59 (5)	0.69 (0.51 – 0.80)	328.03	18.11	50.20	23 (39%)	23 (39%)	32 (54%)
Mental Health	59 (5)	0.63 (0.44 – 0.76)	127.84	11.31	31.35	8 (14%)	28 (47%)	34 (58%)

[a] 1 outlier case removed

[b] values based on standard scores on a 0 to 100 scale

[c] 2 outlier cases removed

Abbreviations: SEM = standard error of measurement; SDC = smallest detectable change
